# Protein with negative surface charge distribution, Bnr1, shows characteristics of a DNA‐mimic protein and may be involved in the adaptation of *Burkholderia cenocepacia*

**DOI:** 10.1002/mbo3.1264

**Published:** 2022-02-06

**Authors:** Ruth Dennehy, Niamh Duggan, Simon Dignam, Sarah McCormack, Eugene Dillon, Jessica Molony, Maria Romano, Yueran Hou, Laura Ardill, Matthew V. X. Whelan, Zuzanna Drulis‐Kawa, Tadhg Ó'Cróinín, Miguel A. Valvano, Rita Berisio, Siobhán McClean

**Affiliations:** ^1^ Centre of Microbial Host Interactions Institute of Technology Tallaght Dublin Ireland; ^2^ School of Biomolecular and Biomedical Science University College Dublin Dublin Ireland; ^3^ UCD Conway Institute of Biomolecular and Biomedical Research University College Dublin Belfield, Dublin Ireland; ^4^ Institute of Biostructures and Bioimaging National Research Council Naples Italy; ^5^ Department of Pathogen Biology and Immunology, Institute of Genetics and Microbiology University of Wroclaw Wroclaw Poland; ^6^ School of Medicine, Dentistry and Biomedical Sciences, Wellcome‐Wolfson Institute for Experimental Medicine Queen's University Belfast Belfast UK

**Keywords:** bacterial adaptation, *Burkholderia cenocepacia*, chronic infection, cystic fibrosis, DNA‐mimic protein, protein–protein interaction

## Abstract

Adaptation of opportunistic pathogens to their host environment requires reprogramming of a vast array of genes to facilitate survival in the host. *Burkholderia cenocepacia*, a Gram‐negative bacterium with a large genome of ∼8 Mb that colonizes environmental niches, is exquisitely adaptable to the hypoxic environment of the cystic fibrosis lung and survives in macrophages. We previously identified an immunoreactive acidic protein encoded on replicon 3, BCAS0292. Deletion of the BCAS0292 gene significantly altered the abundance of 979 proteins by 1.5‐fold or more; 19 proteins became undetectable while 545 proteins showed ≥1.5‐fold reduced abundance, suggesting the BCAS0292 protein is a global regulator. Moreover, the ∆BCAS0292 mutant showed a range of pleiotropic effects: virulence and host‐cell attachment were reduced, antibiotic susceptibility was altered, and biofilm formation enhanced. Its growth and survival were impaired in 6% oxygen. In silico prediction of its three‐dimensional structure revealed BCAS0292 presents a dimeric β‐structure with a negative surface charge. The ΔBCAS0292 mutant displayed altered DNA supercoiling, implicated in global regulation of gene expression. Three proteins were identified in pull‐downs with FLAG‐tagged BCAS0292, including the Histone H1‐like protein, HctB, which is recognized as a global transcriptional regulator. We propose that BCAS0292 protein, which we have named **B**urkholderia **n**egatively surface‐charged **r**egulatory protein 1 (Bnr1), acts as a DNA‐mimic and binds to DNA‐binding proteins, altering DNA topology and regulating the expression of multiple genes, thereby enabling the adaptation of *B. cenocepacia* to highly diverse environments.

## Graphical abstract

**B**urkholderia **n**egatively surface‐charged **r**egulatory protein 1 (Bnr1) is an acidic protein expressed by *Burkholderia cenocepacia* that is upregulated in the stationary phase; in response to low oxygen stress; or the hypoxic environment of the chronically infected cystic fibrosis lung. It possesses a negatively charged surface and its deletion dramatically alters the abundance of over a thousand proteins in the cell, with multiple effects on pathogenesis. We show that Bnr1 binds to histone‐like binding proteins and behaves as a DNA‐mimic protein.

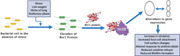

## 1 INTRODUCTION

Pathogenic bacteria experience a broad range of stress conditions within their hosts, including changes in temperature, oxygen levels, pH, nutrient limitation, and antimicrobial molecules. Bacteria respond and adapt to these stresses by reprogramming the expression of large numbers of genes. Environmental opportunistic pathogens, such as *Pseudomonas aeruginosa*, *Acinetobacter baumannii*, *Mycobacterium marinum, Bacillus subtilis, Vibrio vulnificus*, and members of genus *Burkholderia* preferentially colonize environmental niches, including soil and watercourses, and their success as human pathogens relies on their plasticity to adapt to varying conditions (Cullen & McClean, 2015; Hogardt & Heesemann, 2010; Howard et al., 2012; Jeong et al., 2010; Marvig et al., 2014; O'Connor & McClean, 2017; Trunk et al., 2010). Antimicrobial resistance (AMR) ranks among the most important threats to human health. Among the most problematic AMR bacteria, the so‐called ESKAPE pathogens (acronym for *E*. *faecium, Staphylococcus aureus, Klebsiella pneumoniae, A. baumannii, P. aeruginosa* and *Enterobacter* spp), are opportunistic pathogens that are highly adaptable in their ability to colonize a diverse range of niches including the human host (De Oliveira et al., 2020). Insights into the adaptability of these successful opportunistic pathogens will enable the development of alternatives to antimicrobial therapies. Other examples of exquisitely adaptable pathogens which colonize very diverse ecological niches ranging from contaminated soils, water sources, and pharmaceutical plants to the human lung include the Gram‐negative genus *Burkholderia*. These bacteria possess large genomes (7.6–8 .5 Mb; Garcia‐Romero & Valvano, 2020; Holden et al., 2009) and have enormous genetic potential for adaptation within the host. Within this genus, *Burkholderia cepacia* complex (Bcc) is a group of closely related bacteria that causes opportunistic chronic infections in people with cystic fibrosis (CF; Mahenthiralingam et al., 2008). The complex currently comprises at least 22 validly named species (Bach et al., 2017; Depoorter et al., 2020) that are highly resistant to different classes of antibiotics (Zhou et al., 2007). Some members of the complex, notably *B. cenocepacia* and *B. multivorans* have been shown to survive in macrophages and to attach to human epithelial cells (Caraher et al., 2007; Cullen et al., 2017; Saini et al., 1999; Schmerk & Valvano, 2013; Valvano, 2015). Further, *B. cenocepacia* can persist in 6% oxygen through a coregulated 50‐gene cluster of genes, designated the low‐oxygen activated (*lxa*) locus (Sass et al., 2013) which we have shown to be upregulated during chronic infection in response to the hypoxic environment of the CF lung (Cullen et al., 2018).

**Figure 1:**
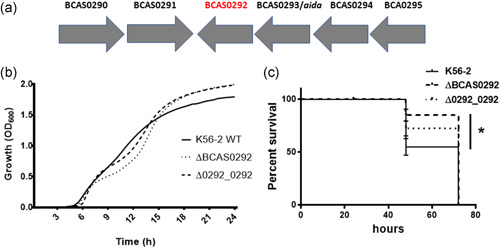
Effect of BCAS0292 deletion on virulence. (a) BCAS0292 gene cluster. Genetic organization of the gene cluster encoding the BCAS0292/Bnr1 and AidA proteins in *Burkholderia cenocepacia* J2315 and K56‐2. The direction of transcription of each gene is indicated by the gray arrows. BCAS gene designations are according to the annotation of the *B. cenocepacia* J2315 genome (Holden et al., 2009). (b) Growth of K56‐2, ∆BCAS0292, and ∆0292_0292 strains at 37°C. (c) Effect of BCAS0292 gene deletion on virulence. Kaplan–Meier plot showing *Galleria mellonella* survival following exposure 2 CFU of K56‐2 WT, ΔBCAS0292 mutant or Δ0292_0292 complement strain. Data were collated from three independent experiments. *Statistical difference between K56‐2 WT and Δ*BCAS0292*; *p* = 0.0036. CFU, colony‐forming unit; WT, wild‐type

Previously, we identified an immunogenic protein encoded by the BCAS0292 gene in *B. cenocepacia* using serum from Bcc‐colonized people with CF (Shinoy et al., 2013). This gene encodes a hypothetical protein and is located within a virulence gene cluster on replicon 3 of the *B. cenocepacia* genome (Figure 1), which also includes *aidA* (BCAS0293). The *aidA* gene resides in the same operon as BCAS0292 and encodes a protein that is required for virulence in *C. elegans* (Huber et al., 2004). Both BCAS0292 and AidA proteins have been classified as PixA proteins, a poorly understood group of inclusion body proteins associated with cells in the stationary phase (Goetsch et al., 2006; Winsor et al., 2008). Both BCAS0292 and *aidA* are the most highly regulated genes in the quorum‐sensing mutants, ∆*cepR*, and ∆*cepRcciIR* (O'Grady et al., 2009). Both genes are also involved in adaptation to the host in a rat chronic infection model (O'Grady & Sokol, 2011). Further, transcripts of both genes are upregulated under low oxygen conditions, and BCAS0292 is also the most upregulated gene in stationary phase growth, suggesting it may be involved in the stress response of *B. cenocepacia* during anoxic and low nutrient conditions (Sass et al., 2013). Overall, these studies suggest a role for the BCAS0292‐encoded protein in the stress response of *B. cenocepacia*; however, its specific function remained unknown. In this study, we constructed a ∆BCAS0292 deletion mutant strain to investigate its role in *B. cenocepacia* pathogenesis. We demonstrate that loss of BCAS0292 alters the abundance of more than a thousand proteins and causes a range of pleiotropic effects, suggesting the protein is involved in the global regulation of gene expression. We also provide evidence indicating the protein has surface characteristics comparable to known DNA mimic proteins and propose that BCAS0292 functions as a DNA mimic by interacting with DNA‐binding proteins and altering the state of DNA supercoiling and have dubbed the protein **B**urkholderia **n**egative surface charged **r**egulatory protein 1 (Bnr1).

## 2 Experimental procedures

### 2.1 Bacterial and epithelial cell culture maintenance

*B. cenocepacia* strain K56‐2 was obtained from the Belgian Coordinated Collection of Microbes/Laboratorium voor Microbiologie, Ghent (BCCM/LMG), University of Ghent, Belgium, and was routinely plated on to *B. cepacia* selective agar (Henry et al., 1997). Bacteria were routinely grown in Luria‐Bertani (LB broth) at 37°C with orbital agitation (150 rpm). The CFBE41o^−^ cell line, which is homozygous for the ΔF508 mutation, was maintained in fibronectin/vitrogen coated flasks containing minimal essential medium with 10% fetal bovine serum, 1% penicillin/streptomycin, 1% l‐glutamine, and 1% nonessential amino acids and incubated in 5% CO_2_ environment at 37°C (Wright et al., 2011).

### 2.2 Mutagenesis of *B. cenocepacia* K56‐2 and complementation

Unmarked, nonpolar gene deletions were constructed as previously described (Flannagan et al., 2008). The deletion of BCAS0292 was confirmed by DNA sequencing of the polymerase chain reaction (PCR) product spanning the deletion site. To complement *B. cenocepacia* K56‐2_ΔBCAS0292, wild‐type (WT) BCAS0292 was amplified from *B. cenocepacia* K56‐2 (see primer pairs in Table A1, A2), digested with the restriction enzymes *Nde*I and *Xba*I, and ligated into similarly digested pMH447. The complementation plasmid was introduced into the mutant by conjugation. Once transferred into the target mutant strain the complementation vector integrates into the genome at aminoglycoside efflux genes (*BCAL1674‐BCAL1675*; Hamad et al., 2010). As before, pDAISce‐I was then introduced, resulting in the replacement of BCAL1674‐1675 by BCAS0292. The complementation of BCAS0292 was confirmed by PCR and then confirmed by phenotype analysis. The mutant and complement strains were cured by growth on LB agar enriched with 5% (wt/vol) sucrose. The growth of all the strains was measured by inoculating LB broth in a 96‐well plate pre‐equilibrated in a BioTek Synergy H1 Hybrid reader at 37°C with log growing cultures in replicates of six. Growth was monitored by measuring the OD_600_ every 30 min for 24 h.

### 2.3 *Galleria mellonella* infection model

Log‐phase grown bacteria were suspended to a starting OD_600nm_ in 0.1 in sterile phosphate buffer saline (PBS) and serially diluted to 10^−7^. Each dilution (20 µl) was injected into the hindmost left proleg of *G. mellonella* larvae using a sterile Terumo 0.3‐ml syringe (10 larvae per group; Costello et al., 2011). An equal volume of each dilution was also plated onto LB agar counted at 48 h to accurately quantify bioburden injected. Ten larvae injected with PBS alone served as controls. Larvae were incubated at 37°C and examined for survival at 24, 48, and 72 h following injection.

### 2.4 Cloning and expression of recombinant BCAS0292

The forward primer was designed to contain the “CACC” sequence at the beginning of the primer to introduce a 5′ overhang allowing directional cloning of BCAS0292 into the pET100/d‐Topo vector in frame with the N‐terminal 6xHis tag. PCR amplification was carried out using HotStar Taq polymerase (Qiagen) and amplicons cloned into the pET100/d‐Topo vector as previously described (Dennehy et al., 2017). Each transformation reaction was spread onto prewarmed selective plates containing 50 μg/ml of ampicillin and incubated overnight at 37°C and transformants identified by PCR. *Escherichia coli* BL21 Star™ (DE3) One Shot® cells (Thermo Fisher Scientific) were used for protein expression (Dennehy et al., 2017). Following the confirmation of recombinant BCAS0292 (rBCAS0292) expression in a pilot study, 1 L cultures of BL21 cells transformed with the expression plasmid, were grown in LB‐ampicillin (100 μg/ml), and induced with isopropyl ß‐d‐1‐thiogalactopyranosideI (IPTG) at a final concentration of 1 mM overnight at 25°C. Bacteria were then pelleted using centrifugation at 2500 g for 10 min. Supernatants were discarded, pellets weighed and stored at −80°C.

### 2.5 Recombinant BCAS0292 purification

Purification of rBCAS0292 was carried out as previously described (Dennehy et al., 2017). Ice‐cold lysis buffer containing 6 M Guanidine‐HCL, 100 mM sodium phosphate, 10 mM Tris‐HCL, (pH 8), and 1X EDTA‐free protease inhibitor was added to bacterial pellets at a volume of 30 ml/7 g of pellet. Cells were resuspended in lysis buffer and lysed on ice with a cell disruptor (maximum output, two 30‐s pulses). Cell debris and any remaining intact cells were pelleted using centrifugation at 4800*g* for 35 min. Supernatants were filtered through a 0.45 μM filter and incubated with a rotating HisPur Ni‐NTA column (Thermo Fisher Scientific), which was pre‐equilibrated with Buffer A, for 1 h at 4°C. Unbound proteins were removed by a series of washes and bound protein was subsequently eluted in two steps with 250 mM and 300 mM imidazole. Following purification, the protein concentration of each fraction was determined using the Bradford assay. Protein purity was determined by sodium dodecyl sulfate–polyacrylamide gel electrophoresis (SDS‐PAGE).

### 2.6 Attachment of bacteria to epithelial cells

To quantify the attachment of Bcc or BCAS0292‐expressing *E. coli* strains to lung epithelial cells, CFBE41o^−^ cells were seeded in coated chamber slides 24 h before the 30 min incubation of individual bacterial strains at an MOI of 50:1, as previously described (Dennehy et al., 2017). In the case of studies with recombinant *E. coli* strains, rBCAS0292 expression was induced in all strains (*E. coli* BL21, *E. coli* BL21_BCAS0292) with 1 mM IPTG for 3 h before incubation with epithelial cells. Unattached bacteria were removed by washing with sterile PBS four times for 5 min each. The CFBE41o^−^ cells and attached bacteria were fixed in 3% paraformaldehyde (pH 7.2) for 10 min at room temperature (RT). Cells were gently washed once with PBS and then blocked in PBS containing 5% bovine serum albumin (BSA) for 1 h at RT. Cells were then incubated overnight at 4°C in PBS containing 1% BSA and either a rabbit anti‐Bcc antibody (1:800 dilution) or an anti‐*E. coli* fluorescein isothiocyanate (FITC)‐conjugated antibody (1:100 dilution). The following day cells were washed three times with PBS before the addition of FITC‐conjugated anti‐rabbit antibody (1:500 dilution), in the case of Bcc strains, for 1 h in the dark. The secondary antibody was removed, and cells were washed twice for 5 min each with PBS. Cells were then counterstained with 4′,6‐diamidino‐2‐phenylindole and phalloidin conjugated to Alexa fluor 568 (5 U/ml) for 15 min in the dark at a concentration of 1 μg/ml in PBS. A coverslip was applied to each chamber before bacteria and cells were visualized using confocal microscopy. The bacterial attachment was counted in 20 randomly selected fields for each strain and values were expressed as the number of bacteria/100 cells.

### 2.7 Membrane permeability

Membrane permeability was determined by measuring the accumulation of the fluorescent dye H33342 as outlined previously (Dennehy et al., 2017). Briefly, strains were aliquoted in replicates of eight in a 96‐well plate and pre‐equilibrated in a BioTek Synergy H1 Hybrid reader at 37°C. H33342 (10 μM) was added to each well and the plates were incubated for 90 min at 37°C with fluorescent readings being taken every minute (*λ*_ex_ = 355 nm, *λ*_em_ = 460 nm). The mean fluorescence of eight replicates relative to the control fluorescence at each time point and at zero time was used to determine the dye accumulation in each strain.

### 2.8 Biofilm formation

Biofilm formation was determined as outlined previously (Caraher et al., 2007) by inoculation of 100/well of mid‐log phase cultures (OD_600_ = 0.6) into microtiter plates in replicates of eight. The plates were incubated at 37°C for 24, 48, or 72 h, rinsed five times with water to remove unadhered bacteria, and air‐dried, airdried, and stained with crystal violet (0.125%) for 30 min at RT. The plates were then rinsed again five times and the dye dissolved with 95% ethanol. The contents of the wells were then transferred to a new polystyrene microtitre plate and absorbance was determined at 590 nm (Tecan Plate Reader; Alpha technologies). Biofilm formation was defined as the wells showing A590 > 0.05.

### 2.9 Whole proteome analysis

To extract whole cell lysates were extracted from 18 h stationary phase cultures of three biological replicates of K56‐2 WT and the ΔBCAS0292 mutant strains. Bacterial cells were pelleted with centrifugation of 6000*g* for 10 min. Ice‐cold lysis buffer containing 8 M urea, 25 mM Tris‐HCL, 10 mM 1,4‐dithiothreitol (DTT), and 1X EDTA‐free protease inhibitor cocktail (pH 8.6) was added to pellets, using a volume of 8 ml/g bacterial pellet. Pellets were resuspended and cells lysed using a cell‐disruptor probe set with an output power of 16, using four 30‐s bursts on ice. Lysates were then centrifuged at 14,000*g* for 15 min, supernatants were transferred to fresh tubes, and a Bradford assay was used to determine the protein concentration of each sample. DTT (1 M) was added to each supernatant (10 μl/ml lysate) and incubated at 56°C for 30 min followed by 1 M iodoacetamide (55 μl/ml lysate) which was incubated in darkness at RT for 20 min. Lysates were then dialyzed in dialysis tubing with a cut‐off of 3.5 kDa against 100 mM ammonium bicarbonate overnight with stirring at 4°C then the ammonium bicarbonate was replaced with fresh solution and incubated for a further 6 h. Trypsin (400 ng/μl) was added to dialyzed protein (5/100 μl protein) and tubes were incubated overnight at 37°C. Aliquots (20 μl) from each sample were placed in fresh tubes and samples were dried in a speedy vac using medium heat and resuspended in 20 μl resuspension buffer containing 0.5% TFA in MilliQ water. Tubes were sonicated for 2 min and centrifuged briefly. ZipTips (Millipore) were wetted with 10 µl of a wetting solution containing 0.1% TFA in 80% acetonitrile slowly five times. Equilibration solution (10 µl) containing 0.1% TFA in MilliQ water was aspirated and dispensed into the ZipTip, five times before 10 µl of the resuspended sample was then aspirated and dispensed slowly into the ZipTip, 15 times. Then 10 µl of the washing solution containing 0.1% trifluoroacetic acid (TFA) in MilliQ water was aspirated into the ZipTip and dispensed to waste. This step was repeated five times before 10 µl of the elution solution containing 0.1% TFA in 60% acetonitrile was aspirated and dispensed from the ZipTip to a fresh 1.5 ml tube six times. Samples were then dried under medium heat and resuspended in 15 μl of loading buffer containing 0.05% TFA in 2% acetonitrile. Tubes were sonicated for 2 min, centrifuged briefly and samples were transferred into fresh vials and 3 μl (1 μg protein) used for Q‐Exactive analysis. All biological replicate and technical replicate samples were analyzed on a Thermo Scientific Q Exactive mass spectrometer (MS) connected to a Dionex Ultimate 3000 (RSLCnano) Chromatography System. Each sample was loaded onto an EASY‐Spray PepMap RSLC C18 Column (50 cm × 75 μm; Thermo Fisher Scientific), and was separated by an increasing acetonitrile gradient over 120 min at a flow rate of 250 nl/min. The MS was operated in positive ion mode with a capillary temperature of 220°C, and with a potential of 2500 V applied to the frit. All data were acquired with the MS operating in automatic data‐dependent switching mode. A high resolution (140,000) MS scan (300–2000 Da) was performed using the Q‐Exactive to select the 15 most intense ions before MS/MS analysis using higher‐energy collisional dissociation. Protein identification and label‐free quantitative (LFQ) analysis was conducted using MaxQuant (version 1.2.2.5, https://maxquant.org/) supported by the Andromeda database search engine to correlate MS/MS data against the *B. cenocepacia* strain J2315 Uniprot database (UP000001035) and Perseus to organize the data (version 1.4.1.3; Cox & Mann, 2008; Cox et al., 2011; Tyanova Temu, & Cox, 2016a; Tyanova, Temu, & Sinitcyn, 2016b). For protein identification, the following search parameters were used: precursor‐ion mass tolerance of 1.5 Da, fragment ion mass tolerance of 6 ppm with cysteine carbamidomethylation as a fixed modification, and a maximum of two missed cleavage sites allowed. False discovery rates (FDR) were set to 0.01 for both peptides and proteins and only peptides with a minimum length of six amino acids were considered for identification. Proteins were considered identified when a minimum of two peptides for each parent protein was observed. LFQ intensities measured for individual runs were grouped based on their experimental treatment. The data were log2 transformed and an analysis of variance (ANOVA) (*p* < 0.05) was performed between the control and individual treatment samples to identify differentially abundant proteins. A log2 fold change ≥ 1.5 and log2 fold change ≤−1.5 with adjusted *p* < 0.05 were considered. Proteins with statistically significant differential expression were extracted and these were used to generate maps of expression. To improve the visual representation of differentially abundant proteins, mean values were generated for each treatment and used to build the maps. A qualitative assessment was also conducted. This involved the identification of proteins that were completely absent in a specific strain. Those proteins that were completely missing from all replicates of a particular group but present in other groups were determined manually from the data matrix. These proteins are not considered statistically significant as the values for absences are given as not a number which is not a valid value for an ANOVA analysis. However, the complete absence of a protein from a group may be biologically significant and these proteins are reported as qualitatively differentially expressed.

### 2.10 Bacterial cell shape analysis

Cell shape was examined according to published methods (Shiomi et al., 2008). Cells were grown at 37°C in LB overnight before reinoculation and culture to achieve log growth (OD_600_ of 0.5–0.7). Optical sectioning was performed with an Olympus FV‐10 ASW confocal microscope using a Plan Apo X60 1.40 oil immersion objective lens and Olympus FLUOVIEW Ver.3.1 software. Section images were captured along the *z*‐axis at 0.2‐μm intervals.

### 2.11 Circular dichroism spectroscopy

Circular dichroism measurements were carried out using a Jasco J‐810 spectropolarimeter equipped with a Peltier temperature control system (Model PTC‐423‐S). The molar ellipticity per mean residue, [*θ*](deg cm^2^ × dmol^−1^), $\left[\theta \right]({\text{deg cm}}^{2}\times {\text{dmol}}^{-1})$ was calculated from the equation [*θ*] = [*θ*]obs × mrw × (10 × *l* × C)^−1^, $\left[\theta \right]=\left[\theta \right]\text{obs}\times \text{mrw}\times {\left(10\times l\times C\right)}^{-1},$ where [*θ*]obs is the ellipticity measured in degrees, mrw is the mean residue molecular mass (105.3 Da), *C* is the protein concentration in g × L^−1^ and l is the optical path length of the cell in centimeter. Far‐UV spectra (190–260 nm) of recombinant BCAS0292 protein were recorded at 293 K using a 0.1 cm optical path‐length cell, with a protein concentration of 0.2 mg ml^−1^. For thermal denaturation experiments, ellipticity was monitored at 222 nm with a temperature slope of 1°C/min from 20 to 100°C. Melting temperature (*T*_m_) was calculated from the maximum of the first derivative of the unfolding curves.

### 2.12 Light scattering analysis

Purified rBCAS0292 was analyzed by size exclusion chromatography (SEC) coupled with light scattering (LS) using a DAWN MALS instrument and an Optilab rEX (Wyatt Technology). Eight hundred micrograms of purified rBCAS0292 protein were loaded into column S75 16/60 (GE Healthcare), equilibrated in 50 mM Tris‐HCl, 150 mM NaCl, 5% glycerol, pH 7.5 and. The online measurement of the intensity of the Rayleigh scattering as a function of the angle as well as the differential refractive index of the eluting peak in SEC was used to determine the weight‐average molar mass (MW) of eluted protein, using the Astra 5.3.4.14 (Wyatt Technologies) software.

### 2.13 Homology modeling

The homology model structure of BCAS0292 was built after consensus‐based sequence comparison using HHPRED. The best model template was identified as the structure of the PixA inclusion body protein from *Burkholderia cenocepacia* (PDB code 4lzk, sequence identity 23.2%, *E*‐value: 7.2e−38). Using this alignment, the homology model was built using the program MODELLER (Bitencourt‐Ferreira & De Azevedo, 2019). As a validation tool, an independent model was computed using i‐TASSER (J. Yang & Zhang, 2015) and the two models were compared. The meta‐threading approach LOMETS was used to retrieve template proteins of similar folds from the PDB library. The program SPICKER was used to cluster the decoys based on the pair‐wise structure similarity. The best confidence model presented a *C*‐score −0.34 (J. Yang & Zhang, 2015). The two approaches provided consistent models, with root‐mean‐square deviation computed on Cα atoms of 1.5 Å. Based on the estimated local accuracy of the model computed by i‐TASSER, the C‐terminal 10 residues were removed from the model. The electrostatic potential surface was computed using the program Chimera (Z. Yang et al., 2012).

### 2.14 Chloroquine gel electrophoresis

*B. cenocepacia* strains were transformed with the pDA‐12 reporter plasmid and cultured on plates containing tetracycline (150 μg/ml), ampicillin (100 μg/ml), and polymyxin B (50 μg/ml). The reporter plasmids were extracted from either log or stationary phase cultures using a Qiagen Miniprep Kit (Thermo Fisher Scientific). The plasmids were analyzed on chloroquine gels (1.5% agarose, 10 μg/ml chloroquine) separated for 24 h at 100 V, 150 mA (Scanlan et al., 2017). The gels were washed 8–12 times over 8 h to remove chloroquine, before staining with ethidium bromide (10 μg/ml) for 2 h. The gel was washed for 1 h before imaging under UV light (Scanlan et al., 2017).

### 2.15 FLAG‐pulldown assay to identify BCAS0292 binding partners

The BCAS0292 gene was cloned into a pT7‐FLAG‐2 plasmid (Sigma‐Aldrich) and transformed into *E. coli* BL21 STAR (DE3) One Shot cells (Invitrogen) for expression. Expression of the recombinant FLAG‐BCAS0292 protein on induction with 1 mM IPTG for 24 h was confirmed using SDS‐PAGE. For the preparation of the bait lysates, *E. coli* BL21 cells transformed with FLAG‐tagged BCAS0292 plasmid were incubated in the presence of 1 mM IPTG at 37°C for 2 h and overnight at RT without shaking. Prey lysates were prepared from K56‐2 WT cells grown to OD_600_ 1.2 at 37°C at 200 rpm. Cells were centrifuged and pellets washed in PBS. The prey lysates pellets were also washed in Tris buffer saline (TBS) (5 ml) three times with centrifugation. Pellets were lysed in 50 mM Tris HCl, 150 mM NaCl, 1 mM EDTA, 1% Triton with protease inhibitor cocktail and DNAse (1 IU) and sonicated for 10 pulses on ice. Cell debris was pelleted by centrifugation at 4800 g (30 min) and lysates were transferred to fresh tubes and stored at −20°C until use. Anti‐FLAG resin was equilibrated in TBS and washed five times before adding to all tubes. The bait cell lysate was immobilized by adding to bait control and bait:prey tubes. TBS was added to the prey control tube. Tubes were incubated at 4°C with shaking for 4 h. The tubes were centrifuged and the resin was washed five times with TBS. The prey cell lysates were added to prey control and bait:prey tubes and TBS added to bait controls and incubated at 4°C overnight before centrifuging and washing with TBS five times. Pull‐down proteins were eluted in 2X sample buffer (10% SDS, 20% glycerol, 125 mM Tris [pH 6.8], 0.004% bromophenol blue) and boiled for 3 min before loading on 12% SDS‐PAGE gels. This was performed three times with independent preparations of prey and bait from independent cultures on each occasion. Protein bands of interest were manually excised from the gels and in‐gel trypsin digestion was performed (Shevchenko et al., 2002) followed by Zip‐tip clean up and identified in technical triplicates with a Thermo Scientific Q Exactive Mass Spectrometer connected to a Dionex Ultimate 3000 RSLCnano chromatography system. Proteins were separated on a C18 column (C18RP Reposil‐Pur; 100 × 0.075 mm × 3 μm) over 60 min at a flow rate of 250 nl/min with a linear gradient of increasing ACN from 1% to 27%. The MS was operated in data‐dependent mode; a high resolution (70,000) MS scan (300–1600 m/z) was performed to select the 12 most intense ions and fragmented using high energy C‐trap dissociation for MS/MS analysis.

Raw data from the Q‐Exactive was processed using MaxQuant (Cox & Mann, 2008; Tyanova Temu, & Cox, 2016a; version 1.6.3.4) incorporating the Andromeda search engine (Cox et al., 2011). MS/MS spectra were matched against Uniprot *B. cenocepacia* database (UP000001035) containing 6933 entries and *E. coli* BL‐21 database (UP000002032) containing 4156 entries. All searches were performed using the default setting of MaxQuant, with trypsin as specified enzyme allowing two missed cleavages and a FDR of 1% on the peptide and protein level. The database searches were performed with carbamidomethyl (C) as fixed modification and acetylation (protein N terminus) and oxidation (M) as variable modifications. The label‐free quantification (LFQ) algorithm was used to generate normalized spectral intensities and infer relative protein abundance. Statistical analysis was performed using Perseus software (Tyanova, Temu, & Sinitcyn, 2016b; version 1.6.15.0). To examine changes in protein expression using volcano plots, data were log‐transformed, and missing values were imputed with values from a normal distribution. Quantitative analyses were performed using a Student's *t*‐test to compare bait:prey and prey samples. Proteins with significant changes in abundance (*p* < 0.05) were included in the quantitative results. Data were normalized using *z*‐score and visualized using heat maps.

### 2.16 Statistical analysis

Analysis of virulence data was performed using a log‐rank (Mantel‐Cox) test on the survival curves. Statistical analysis of host cell attachment, biofilm formation, and hypoxic growth and survival were carried out by two‐way ANOVA using Prism software.

## 3 RESULTS

BCAS0292/Bnr1 belongs to the PixA protein family. These are inclusion body proteins first identified in *Xenorhabdus nematophila* which are typically between 173 and 191 amino acids in length (Goetsch et al., 2006). They are elevated in cells transitioning to the stationary phase (Goetsch et al., 2006), but are poorly understood. Bnr1 has a calculated molecular weight of 19.2 kDa, a calculated pI of 4.81, and has 34 homologs in several sequenced *B. cenocepacia* strains, and in *B. anthina*, *B. cepacia*, *B. pyrrocinia*, and other *Burkholderia* species (Winsor et al., 2008). A BlastP search showed that it is not found outside *Burkholderia* (Altschul et al., 1997). The BCAS0292 gene is encoded in a two‐gene operon with *aidA* (O'Grady et al., 2009) (Figure 1a). AidA is also classified as a PixA protein, while it also shares sequence similarity with PixB of *X. nematophila* (Lucas et al., 2018). The *aidA* gene is more widely distributed across *Burkholderia* species with 417 orthologs identified to date (Winsor et al., 2008). AidA also shares a high level of identity with PixB proteins across Gammaproteobacteria, Alphaproteobacteria, Actinobacteria, and species (Lucas et al., 2018). Although Bnr1 is immunoreactive, indicating that it is expressed during human infection, we did not detect AidA in our immunoproteomic experiments (Shinoy et al., 2013). To investigate the potential function of Bnr1 in infection, we constructed a deletion mutant of BCAS0292 in K56‐2, a *B. cenocepacia* strain that is amenable to genetic manipulation and a complemented strain (referred to as ∆BCAS0292 and ∆0292_0292, respectively) and compared their phenotypes to the WT strain. The growth of the mutant and complement strains was comparable to that of the WT K56‐2 strain (Figure 1b).

### 3.1 Virulence of K56‐2 WT and ΔBCAS0292 strains in *G. mellonella* infection model

To examine any potential role in pathogenicity, the virulence of the K56‐2 ΔBCAS0292 mutant strain and the K56‐2 WT strain were compared using the *G. mellonella* wax moth larvae infection model. K56‐2 WT is highly virulent in this model, showing a very low mean LD₅₀ value of 1.7 colony‐forming unit (CFU) at 48 h (Figure 1c) in agreement with previous studies. Deletion of the BCAS0292 gene resulted in a moderate reduction in virulence (Figure 1c; *p* = 0.036) which was partially rescued in the Δ0292_0292 complemented strain, suggesting that Bnr1 contributes to *B. cenocepacia* virulence in this model.

### 3.2 Potential role in host–cell interactions

**Figure 2:**
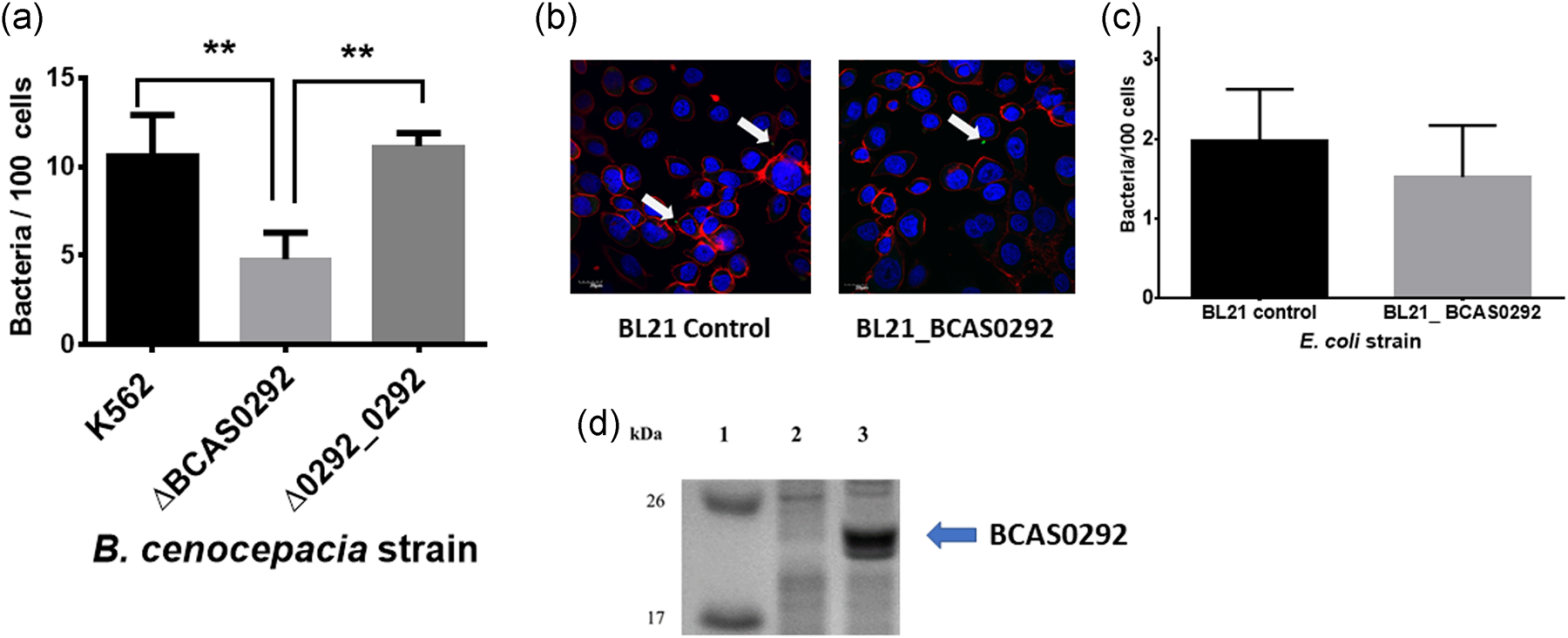
Role of BCAS0292 in epithelial cell attachment. (a) Attachment of K56‐2 WT, ∆BCAS0292 mutant, and ∆0292_0292 complemented strain to CFBE41o^−^ cells at an MOI 50:1. Data represent the mean number of bacteria/100 cells for each strain with 20 fields of view counted per data point in three independent experiments, **p < 0.01. (b) Confocal microscopy images representing the attachment of *Escherichia coli* BL21 control and *E. coli* BL21_BCAS0292 strains to CFBE41o^−^ cells. Bacteria were labeled using a primary anti‐*E. coli* FITC‐conjugated antibody (green) and highlighted with white arrows. Nuclei of CFBE41o^−^ cells were counterstained with DAPI (blue) and actin stained with phalloidin conjugated with Alexa fluor 568. (c) Attachment *E. coli* BL21 control and *E. coli* BL21_BCAS0292 to CFBE41o^−^ cells, using an MOI 50:1. Data represent the mean number of bacteria/100 cells for each strain, with 20 fields of view counted per data point in three independent experiments. (d) Confirmation of expression of Bnr1, following induction in *E. coli* BL21 cells with 1 mM IPTG by Coomassie Blue stained SDS‐PAGE gel (conditions used for Figure 1c). Lane 1: MW marker; Lane 2: *E. coli* BL21 expression control; Lane 3: *E. coli* BL21_BCAS0292 after incubation with IPTG (1 mM) for 2 h determined. DAPI, 4′,6‐diamidino‐2‐phenylindole; FITC, fluorescein isothiocyanate; IPTG, isopropyl ß‐d‐1‐thiogalactopyranosideI; MOI, multiplicity of infection; SDS‐PAGE, sodium dodecyl sulfate–polyacrylamide gel electrophoresis; WT, wild‐type

We have previously shown that other immunogenic proteins, including peptidoglycan‐associated lipoprotein, are involved in attachment to host epithelial cells (Dennehy et al., 2017, McClean et al., 2016). The mutant strain ΔBCAS0292 displayed a 2.2‐fold reduction in attachment to CFBE41o^−^ cells relative to the WT strain (*p* = 0.0073) (Figure 2a). Adhesion was rescued to WT levels in the Δ0292_0292 complemented strain, suggesting a role for the Bnr1 protein in host cell attachment (Figure 2a). Bnr1 is predicted to localize at the inner membrane (PSort prediction tool: https://psort1.hgc.jp/form.html; certainty 0.117). Therefore, to examine if it was directly involved in host cell attachment, the BCAS0292 gene was cloned into *E. coli* BL21 cells, expression induced with 1 mM IPTG and cellular attachment quantified using confocal immunofluorescent microscopy (Figure 2b). *E. coli* binds poorly to lung epithelial cells with roughly two cells attached per 100 epithelial cells (Figure 2c). *E. coli* BL21_BCAS0292 cells showed no increase in attachment to CFBE41o^−^ cells, relative to control BL21 cells, with a mean value of 1.5 bacterial cells per 100 epithelial cells. Expression of Bnr1 protein under these experimental conditions was confirmed by SDS‐PAGE electrophoresis (Figure 2d). While attachment to host cells may require other factors not expressed in *E. coli*, the absence of an effect on host cell attachment of *E. coli* expressing Bnr1 together with the reduction in host cell attachment of the ∆BCAS0292 *B. cenocepacia* mutant suggested that the protein may exert an indirect role in bacterial adhesion to lung epithelial cells.

### 3.3 Analysis of the susceptibility of K56‐2 WT and ΔBCAS0292 to polymyxin B and meropenem

**Figure 3:**
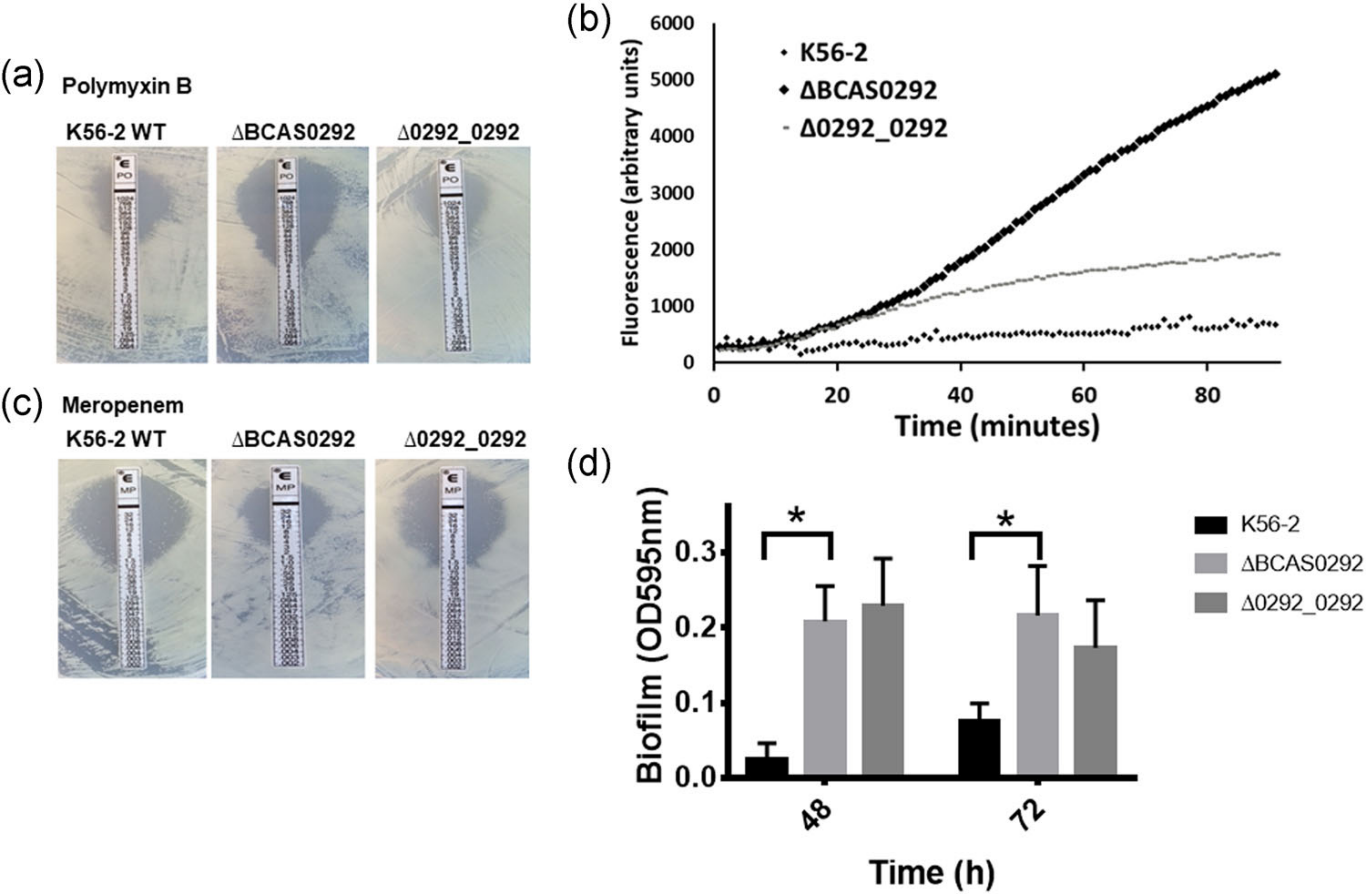
Effect of Bnr1 on antibiotic sensitivity and biofilm formation. Images represent the susceptibility of WT, mutant, and complemented strains to polymyxin B (a), (0.064–1024 μg/ml) or meropenem (b), (0.002–32 μg/ml) using E‐test strips. The data shown are representative of three independent experiments. (c) Accumulation of H33342 fluorescent dye in the ∆BCAS0292 mutant, complement, and wild‐type K5602 strains over 90 min. (d) Bar chart representing biofilm formation of K56‐2 WT, ∆BCAS0292, and ∆0292_0292 after 24, 48, and 72 h, as determined by crystal violet staining. Error bars represent the standard deviation from three independent experiments. *p < 0.0001 represents a significant difference between ∆BCAS0292 and K56‐2 WT strain. WT, wild‐type

Bnr1 expression was previously shown to be increased in response to antibiotics but deletion of the genes encoding Bnr1 and BCAS0293 did not alter antibiotic susceptibility (Sass et al., 2011). To further examine antimicrobial susceptibility, the sensitivity of the ΔBCAS0292 mutant to meropenem and polymyxin B (not previously examined) was measured to determine if the absence of this protein affected susceptibility to either of these antibiotics. The ΔBCAS0292 mutant was eightfold more sensitive to polymyxin B compared to the WT and complemented strains with a minimum inhibitory concentration (MIC) of 12 μg/ml for ΔBCAS0292 compared to 96 μg/ml for the WT and complemented strains (Figure 3a). Polymyxin B targets lipopolysaccharide components (Mares et al., 2009) and destabilizes the outer membrane; suggesting that Bnr1 may play a role in the maintenance of outer membrane integrity. This was supported by the enhanced permeability of the ΔBCAS0292 mutant to the fluorescent dye Hoechst H33342 relative to WT (Figure 3b). Membrane permeability was partially restored to WT levels in the complement strain. In contrast, ΔBCAS0292 was more resistant to the β‐lactam meropenem in comparison to the WT and complemented strains with a MIC of 4 μg/ml for ΔBCAS0292 compared to 1.5 μg/ml for the WT and complemented strains (Figure 3c).

### 3.4 Effect on biofilm formation

Biofilm formation is regulated by the cepIR quorum sensing system, which has been shown to regulate Bnr1 expression (O'Grady & Sokol, 2011). Therefore, we examined whether deletion of the BCAS0292 gene had any effect on biofilm formation. ΔBCAS0292 mutant cells showed significantly increased biofilm formation (*p* < 0.0001) at all time points, which was partially compensated in the complemented strain at 72 h (*p* = 0.0343; Figure 3d), suggesting that the Bnr1 protein repressed biofilm formation (Table 1).

**Table 1:** Total number of differentially expressed proteins between K56‐2 WT and Δ*BCAS0292* strains

Protein groups	Number of differentially expressed proteins
Total number of proteins identified due to altered abundance	2305
Total number of proteins altered by ≥1.5 or absent in all replicates of either strain	979
Proteins unique to K56‐2 WT	19
Proteins unique to Δ*BCAS0292*	9
Proteins significantly downregulated (by ≥1.5‐fold) in the Δ*BCAS0292* strain	545
Proteins significantly upregulated (by ≥1.5‐fold) in the Δ*BCAS0292* strain	406

Abbreviation: WT, wild‐type.

### 3.5 Whole proteome analysis of WT and ΔBCAS0292 strains

**Figure 4:**
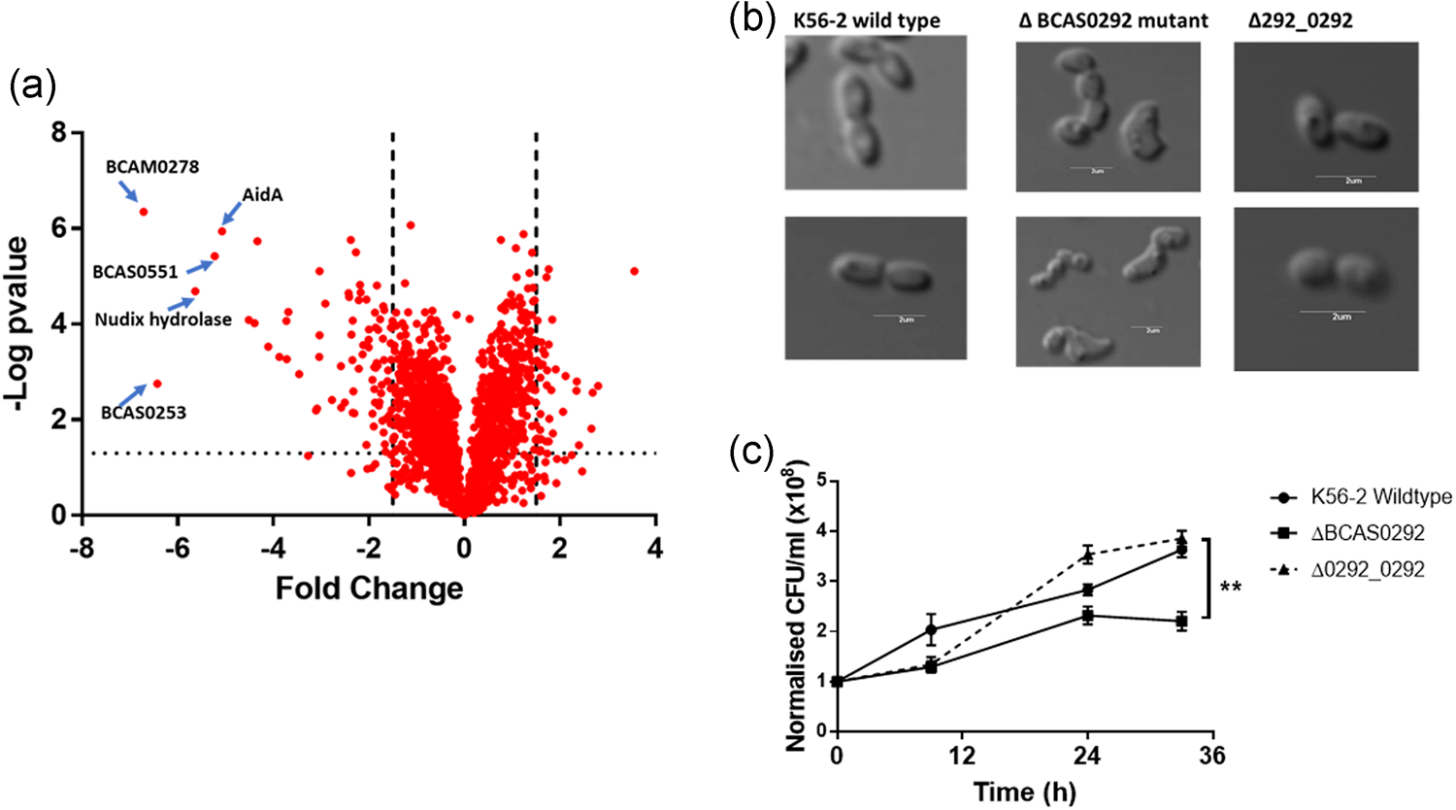
Effect of deletion of the BCAS0292 gene on the proteome and phenotype of *B. cenocepacia* K56‐2. (a) Fold change in protein abundance plotted as a volcano chart of log2 fold change in protein abundance in the ∆BCAS0292 mutant strain relative to WT strain. Points on the negative side of the *X*‐axis represent a reduction in protein abundance. The proteins with the greatest changes in abundance are highlighted with arrows. The dashed line represents the 1.5‐fold change in abundance. (b) Effect of deletion of BCAS0292 on cell morphology as determined by phase‐contrast microscopy. (c) Growth and survival of K56‐2 strain, ∆BCAS0292 mutant, and 0292_0292 complement strain in 6% oxygen. Cells were cultured in a hypoxia chamber at 6% oxygen for up to 48 h. Growth is plotted as OD600 nm. Survival was determined by serially diluting aliquots of cultures that had been incubated at 6% oxygen in a controlled hypoxia chamber, plated at time intervals, and cultured for 48 h in a normoxic environment. WT, wild‐type

The lack of a direct effect on host cell attachment, combined with the apparent repression of biofilm formation and behavior in response to antibiotics suggested that the Bnr1 protein has a regulatory effect. To determine if Bnr1 is a regulatory protein we compared the proteome profiles of the ΔBCAS0292 mutant with WT under stationary phase conditions. Stationary‐phase cultures were used for this experiment since the expression of Bnr1 is maximal in the stationary phase (Sass et al., 2013). Despite there being only a single gene deletion, we identified 2305 proteins showing different abundance between the strains, of which 9 proteins were unique to the ΔBCAS0292 mutant while 19 were unique to K56‐2 WT cells (Table A3). Unique proteins are those which are detected in all replicates of one strain but are undetectable in all four replicates of the comparator. More proteins were showing reduced abundance and the fold‐changes were more extensive than those showing increased abundance (Figure 4a). We identified 406 proteins with significantly increased abundance by ≥1.5‐fold in ΔBCAS0292 while 545 proteins (57%) showed significantly reduced abundance by 1.5‐ to 104‐fold, suggesting Bnr1 is a global positive regulator of protein expression (Figure 4a). Among the proteins showing a reduced abundance in the ΔBCAS0292 mutant were proteins involved in metabolism, virulence, stress responses, and uncharacterized proteins. The fold reduction in protein abundance was substantial: 81 proteins showed reduced abundance (ranging from 3‐ to 104‐fold) in the mutant, and 232 proteins were reduced by over twofold (Table 2). In contrast, the greatest fold increase in abundance was only 11.7, including proteins with roles in metabolic processes, translation, and the stress response (Table S4 at https://doi.org/10.5281/zenodo.5837496). In‐depth analysis of the proteins that had been altered was performed to shed light on the function of Bnr1, the breadth of pleiotropic effects underlined its role as a global regulator.

**Table 2:** Examples of proteins altered by >2‐fold ∆BCAS0292 mutant relative to K56‐2 WT cells

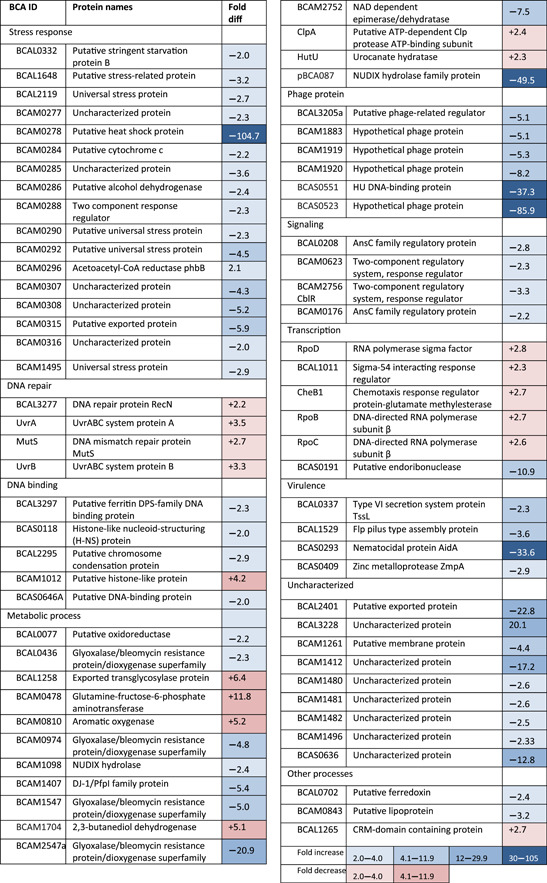

Abbreviation: WT, wild‐type.

Proteins that are unique to the ∆BCAS0292 mutant proteome are likely to be proteins that are repressed by Bnr1. These proteins include RpfR, a receptor for the autoinducer of *Burkholderia* diffusible signaling factor (BDSF) (Table S4 at https://doi.org/10.5281/zenodo.5837496). The majority of proteins that were absent in the mutant (and consequently may rely on the presence of Bnr1 for expression) are proteins with catalytic activity.

The abundance of many proteins with roles in the stress response was reduced in the ΔBCAS0292 mutant, representing 6% of the total number of downregulated proteins in this group. Of particular interest was the reduced abundance of 19 proteins encoded on the low‐oxygen activated (*lxa*) locus (BCAM0272 to 0323) in the mutant strain (Tables 2 and A3). This locus is upregulated under hypoxic conditions and in the stationary phase (Sass et al., 2013) and in sequential clinical *B. cenocepacia* isolates over time of chronic infection (Cullen et al., 2018). Eight *lxa*‐encoded proteins that showed reduced abundance have stress response functions, including a heat shock/α‐crystallin protein (BCAM0278) which was dramatically reduced (104.7‐fold) relative to K56‐2 WT. Five of the six universal stress proteins (USPs) encoded on the *lxa* locus showed reduced abundance in the ∆BCAS0292 mutant strain by up to 4.5‐fold. Other stress response proteins were also reduced in abundance (1.5–2.2‐fold). Four other low‐oxygen coregulated gene loci have been identified (Sass et al., 2013) and five proteins encoded by these *lxa* coregulated genes were also reduced in abundance: BCAM1480–BCAM1482, another USP (BCAM1495), and BCAM1496 (Table 2).

Proteins with roles in various metabolic processes represented over a third of the total number of proteins reduced in abundance, including a plasmid‐encoded NUDIX hydrolase family protein (pBCA087) reduced by 49.5‐fold (Table 2), one of five NUDIX proteins identified with reduced abundance (Table S4 at https://doi.org/10.5281/zenodo.5837496). Nine glyoxalase/bleomycin resistance protein/dioxygenase superfamily proteins were reduced by up to 20.9‐fold (Tables 2 and Table S4 at https://doi.org/10.5281/zenodo.5837496). The proteins unique to the WT strain also had functions in metabolic processes, indicating a global change in the metabolism of these cells in the absence or presence of Bnr1.

A range of virulence proteins, including three T6SS secretion system proteins BCAL0337, tssD, and hcp1, the quorum sensing regulated metalloprotease, ZmpA, nematocidal protein AidA, and an Flp pilus protein (BCAL1529) also showed reduced abundance in the mutant. Bnr1 is identified as a hypothetical protein (Winsor et al., 2008) and 19% of the proteins showing a reduced abundance in the ∆BCAS0292 mutant were also classed as uncharacterized proteins/hypothetical proteins, including BCAL3228 and BCAS0636 (20‐ and 12.8‐fold, respectively). All five PixA proteins in the sequenced J2315 genome (Winsor et al., 2008), showed reduced abundance (up to 17‐fold) suggesting a coregulation or co‐dependence of these uncharacterized proteins. Two of these PixA proteins (BCAM1412 and BCAM1414) are considered orthologs of AidA. Membrane proteins with roles in adhesion and possible roles in structure/transport represented 4% of the total number of proteins reduced in abundance in the mutant strain (Table 2). Among these were proteins associated with roles in adhesion that included putative lipoproteins (BCAM0843 and BCAM0944) and a putative membrane protein (BCAM1261). In addition, two membrane proteins were undetectable in the mutant strain, a lipoprotein (BCAL2166), and a putative membrane protein (BCAM0264) (Table A3).

### 3.6 ∆BCAS0292 mutant shows gross morphological changes

Given the vast number of proteins with altered abundance in the mutant strain, we compared the morphology of the mutant with the WT and complement strains. Unsurprisingly, the ∆BCAS0292 mutant cells had a grossly altered morphology, with a highly irregular cell surface and complete loss of their rod‐like shape (Figure 4b). This was reversed in the complement suggesting that Bnr1 has a direct or indirect role in maintaining cell structure which may contribute to the reduced adherence of this strain to lung epithelial cells.

### 3.7 ∆BCAS0292 mutant has limited growth at 6% oxygen

Given that several proteins encoded on the *lxa* locus showed reduced abundance in the ∆BCAS0292 mutant, we compared the growth of WT and ∆BCAS0292 mutant in 6% oxygen in a controlled hypoxia chamber. The growth (measured as CFU/ml) of the mutant strain was severely reduced under hypoxic conditions (*p* = 0.0138; Figure 4c). In contrast, growth recovered to WT levels in the complemented strain.

### 3.8 Structural analysis of Bnr1 protein

**Figure 5:**
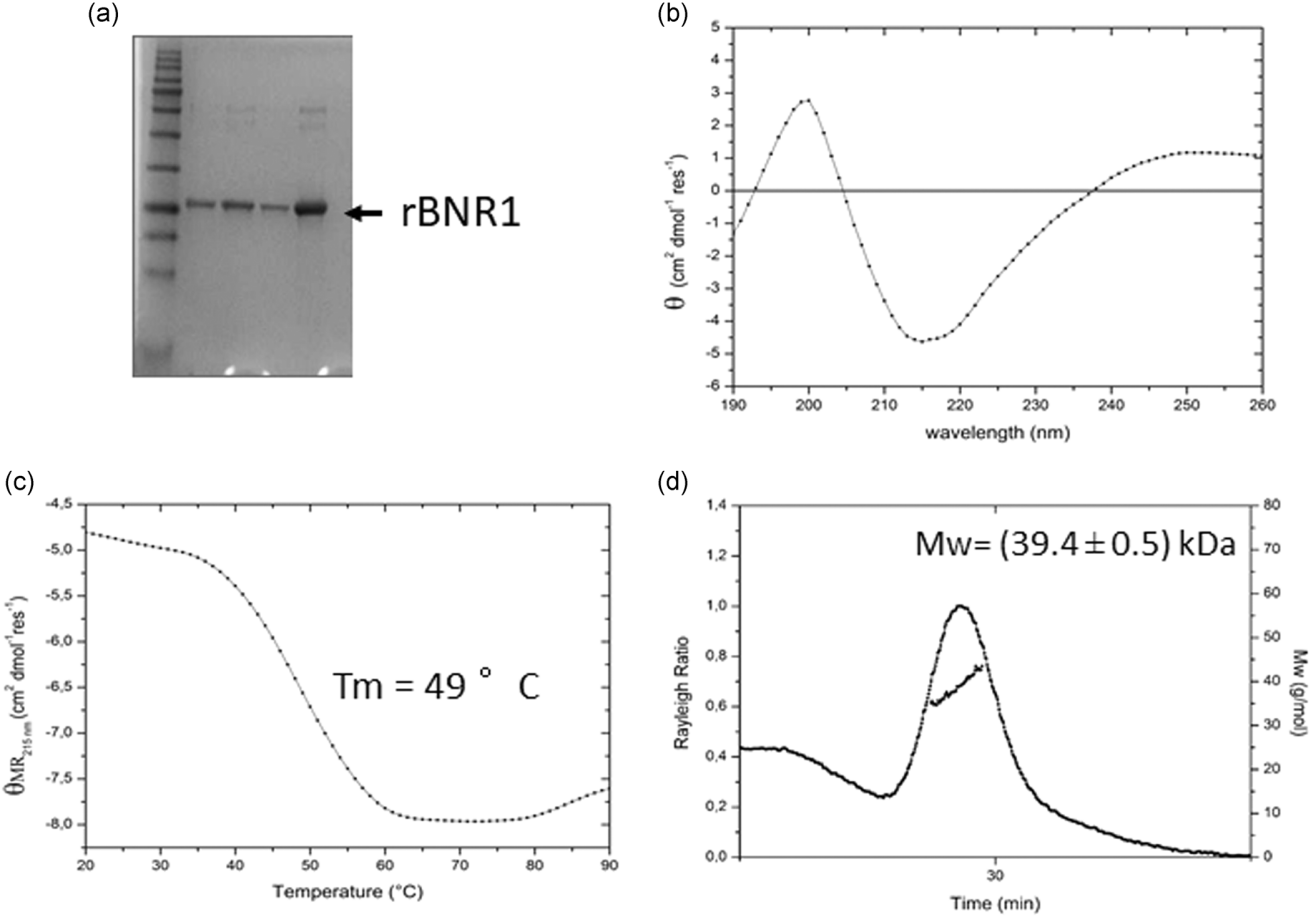
Structural analysis of rBnr1. (a) Size‐exclusion chromatogram and subsequent SDS‐PAGE analysis of purified rBnr1 protein. (b) CD analysis of purified rBnr1 protein to determine the secondary structure at 20°C. A minimum was observed at 215 nm, indicative of a β‐sheet fold. (c) Thermal gradient to determine the *T*_m_ of purified rBnr1, demonstrating a *T*_m_ of 49°C. (d) Light scattering analysis to determine the oligomeric state of rBnr1, which indicated that the protein has a dimeric organization. CD, circular dichroism; SDS‐PAGE, sodium dodecyl sulfate–polyacrylamide gel electrophoresis

**Figure 6:**
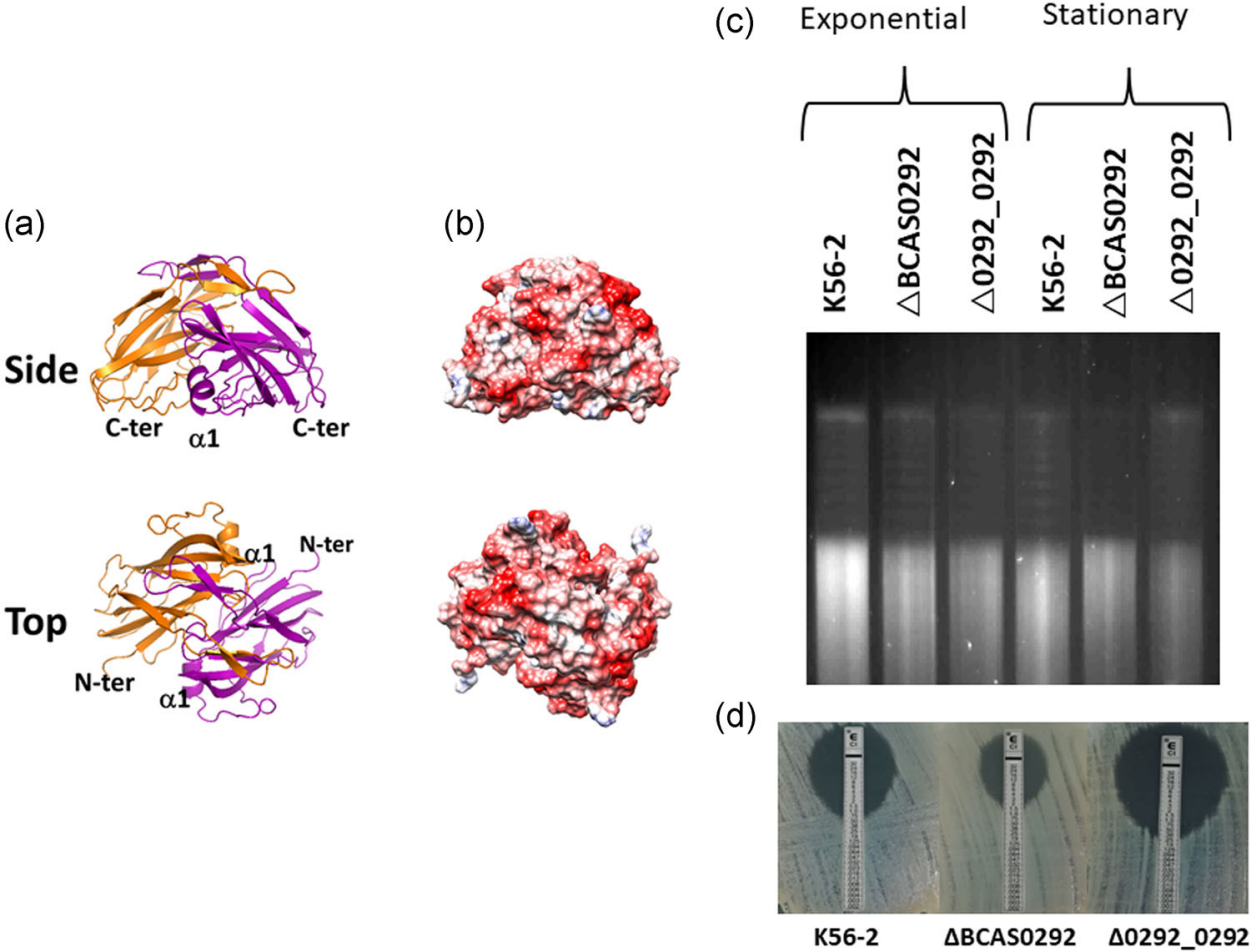
Molecular modeling of Bnr1 and impact on DNA supercoiling (a) Cartoon representation of the predicted three‐dimensional structure of the dimer. Consensus‐based sequence alignment was used to determine the predicted structure using MODELLER software. (b) Analysis of the electrostatic potential of the hypothetical protein. Side view of the predicted dimer model. (c) Comparison of DNA supercoiling of plasmid DNA extracted from *Burkholderia cenocepacia* K56‐2, ∆BCAS0292, and ∆0292_0292 cells cultured to either exponential or stationary phase. Chloroquine‐stained gel image is representative of three independent experiments. (d) Ciprofloxacin susceptibility of K56‐2, ∆BCAS0292, and ∆0292_0292 strains as determined by E‐strips. Images representative of three independent experiments

To help elucidate the function of Bnr1, we analyzed its structural features in solution. Following affinity chromatography purification, His‐tagged Bnr1 was purified by SEC to remove any contaminating proteins or aggregates (Figure 5a). Circular dichroism analysis of rBnr1 showed a peak minimum at 215 nm indicating it has a β‐sheet secondary structure (Figure 5b). The purified protein was highly stable with a melting temperature (*T*_m_) of 49°C, (Figure 5c). LS indicated the oligomeric state of the Bnr1 protein while Rayleigh scattering showed it has a dimeric organization, under reducing and oxidizing conditions, with a molecular weight of 39.4 ± 0.5 kDa (Figure 5d). Despite several attempts, we were unable to obtain crystals of Bnr1, therefore molecular modeling was used to predict the three‐dimensional structure of the protein using consensus sequence alignment with PixA protein BCAM1413 from *B. cenocepacia* as a template (PDB code 4lzk, *E*‐value: 3.5e^−55^; Nocek et al., 2013). Consistent with CD analysis (Figure 5b), the Bnr1 homology model presents a structure with mostly β‐folds, and only one small α helix in the N‐terminal region of the protein (residues 13–21; Figure 6a). Also, in agreement with LS studies (Figure 5d), the molecule has a dimeric organization, where two jelly‐roll‐like monomers are compactly held together (Figure 6a). Electrostatic potential calculations show that the entire surface of the protein has a strong negative charge distribution (Figure 6b), a finding which excludes that Bnr1 regulatory function occurs through interactions with DNA or RNA. Similar electrostatic surface distributions are a distinctive feature of DNA mimicry (Wang et al., 2014).

### 3.9 Bnr1 may function as a DNA mimic protein

Given that Bnr1 had an overall negative surface charge, we hypothesized that it bound to DNA‐binding proteins to mediate its global regulatory activity. To corroborate this hypothesis we examined whether Bnr1 alters DNA supercoiling, an activity associated with global changes in bacterial gene regulation (Scanlan et al., 2017; Shortt et al., 2016) by investigating the impact of the presence of Bnr1 on DNA topology. Bnr1 is upregulated in the stationary phase (Sass et al., 2013), therefore we compared stationary phase cultures with exponential phase cultures for the WT K56‐2, ∆BCAS0292 mutant, and complemented strain. DNA topoisomers are not highly resolved in *B. cenocepacia*, presumably due to the very high levels of restriction‐modification systems expressed in *Burkholderia*. There were no obvious differences in supercoiling in plasmid DNA extracted from exponential phase cultures of the three strains (Figure 6c). In contrast, DNA extracted from stationary phase cultures of the mutant showed a more relaxed DNA topology with fewer highly supercoiled topoisomers at the top of the chloroquine gel relative to WT and complement strains (Figure 6c), indicating that Bnr1 expressed in stationary phase alters DNA supercoiling. Fluoroquinolones target DNA gyrase and topoisomerase IV, enzymes involved in establishing and or relaxing supercoiling. Consequently, any difference in DNA topology may have an impact on the susceptibility to fluoroquinolones between the mutant and WT. The mutant clearly showed a twofold decrease in ciprofloxacin susceptibility, which was reversed in the complemented strain (Figure 6d), further indicating that a lack of Bnr1 expression impacted DNA topology.

### 3.10 Identification of Bnr1 binding partners

**Figure 7:**
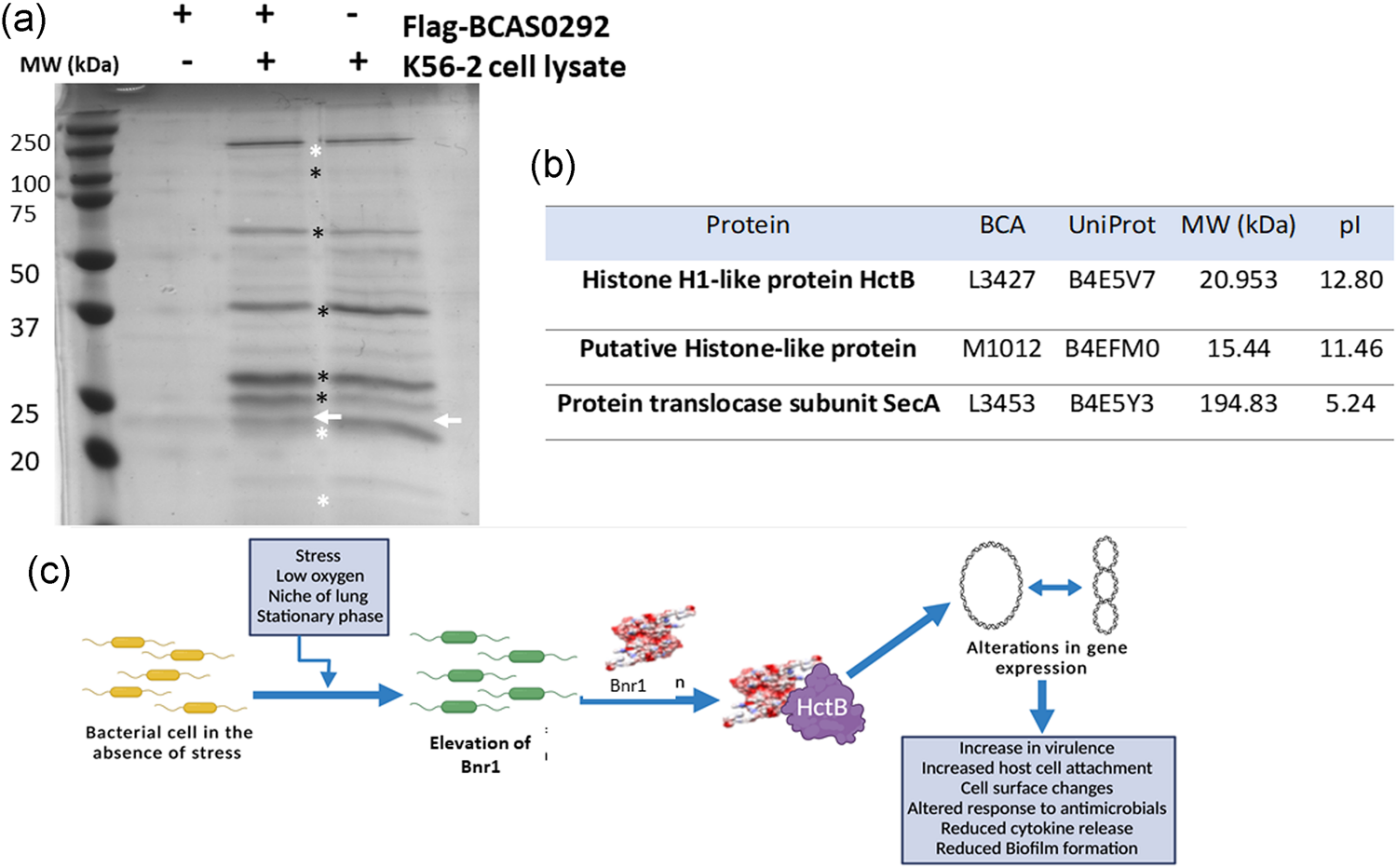
Identification of binding partners and proposed mechanism. (a) Pull‐down analysis of proteins interacting with Flag‐tagged Bnr1. Extracted *Burkholderia cenocepacia* K56‐2 cell lysates were used as prey and Flag‐tagged Bnr1, immobilized on anti‐Flag M2‐agarose affinity resin used as bait, bands of interest at 20/21, 30, 37, 60, 100, 200 kDa, which were analyzed by LC‐MS are highlighted by asterisks; white asterisks highlight bands corresponding to identified binding partners. Bnr1 is highlighted with white arrows. The gel is representative of three independent pull‐down experiments. (b) The three proteins pulled down proteins identified which met our stringency criteria are listed. (c) Schematic of the proposed mechanism of regulation by Bnr1 in response to stress, low oxygen, and stationary phase. Previous work has shown that BCAS0292/Bnr1 is elevated in response to stress, low oxygen, and stationary phase and elevated in late isolates from chronically colonized patients. The present work shows that the absence of Bnr1 reduces the abundance of ≥500 proteins, particularly those including those involved in the stress response, metabolic processes, transcription cell signaling, and virulence. In addition, we have shown that the presence of Bnr1 alters DNA topology, providing a mechanism by which it globally regulates protein expression in response to stress

To identify the proteins that Bnr1 interacted with we performed pull‐down experiments with FLAG‐tagged Bnr1 protein. To ensure reproducible quantification of bait:prey proteins relative to the background, we performed three independent pull‐down experiments on separate days. Bands were selected based on the apparent differences in density in the prey lane relative to the bait:prey and analyzed by MS. Only proteins with at least two unique peptides, found in all technical replicates from at least two independent experiments were selected. Protein enrichments were calculated by comparing protein abundance in the bait:prey versus prey only lanes. Three proteins meeting our stringent selection criteria were reproducibly identified in the prey:bait lanes which were enriched or absent in the two control lanes, (Figure 7a,b; Table S7 at https://doi.org/10.5281/zenodo.5837496). There was a consistent band just below the FLAG‐Bnr1 band, which was identified as HctB, a histone H1‐like protein that is localized at the outer membrane (Winsor et al., 2008). Another putative histone‐like protein was also identified (BCAM1012). Interestingly, the protein translocase subunit, SecA was also identified.

## 4 DISCUSSION

The adaptation of opportunistic pathogens to enhance their fitness in the host and enable their successful colonization requires substantial rearrangement of gene expression. Understanding the processes of bacterial regulation in pathogens that adapt to chronic infection is crucial, particularly in those that are virtually impossible to eradicate. Our initial studies on the *B. cenocepacia* K56‐2 ΔBCAS0292 mutant pointed towards a regulatory role for the Bnr1 protein. The mutant showed reduced attachment to epithelial cells relative to the K56‐2 WT strain but induction of Bnr1 expression had no impact on the adhesion of *E. coli* BL21 cells to CFBE41o^−^ cells, in contrast to our earlier work on peptidoglycan‐associated lipoprotein in which the attachment of *E. coli* BL21 cells to lung cells was enhanced when the recombinant protein was induced (Dennehy et al., 2017). Consistent with this, the dramatic upregulation of BCAS0292 gene in response to hypoxia or stationary phase (Sass et al., 2013), coupled with its dramatic downregulation in the quorum‐sensing mutant strain K56‐2 ∆*cepR* (O'Grady & Sokol, 2011) demonstrated that it is highly responsive and suggested that Bnr1 plays a role in the response of *B. cenocepacia* to extracellular stress. Our work now demonstrates that it has a global regulatory role in the expression of at least 979 proteins, including those involved in stress responses and metabolism. Specifically, the reduced abundance of five out of six USPs and 14 other *lxa*‐encoded proteins in the mutant supports the role of Bnr1 in the regulation of hypoxic stress responses. Further, the impaired growth of the mutant when cultured in 6% oxygen also substantiates a role for Bnr1 in the adaptation of *B. cenocepacia* to hypoxic conditions.

Comparison of the proteomes of the K56‐2 WT and ΔBCAS0292 mutant strains highlight that discrete groups of proteins were affected by the deletion of BCAS0292. A total of eighteen viral proteins were absent or dramatically reduced in the ΔBCAS0292 mutant, suggesting a positive correlation between the expression of specific viral proteins and Bnr1. These include four proteins encoded in a newly identified prophage region (BCAM1879–BCAM1926) not previously reported (Roszniowski et al., 2018). Similarly, all five PixA proteins encoded in *B. cenocepacia* were significantly reduced in the ΔBCAS0292 mutant, indicating that other PixA proteins may require Bnr1 for their expression.

Among the proteins that were unique to the mutant (i.e., undetectable in the WT) was RpfR, the BDSF receptor. RpfR is involved in the synthesis and degradation of cyclic‐di‐GMP, a second messenger which regulates biofilm formation, extracellular proteases, and other virulence phenotypes. Point mutations in different *rpfR* domains resulted in mutants showing increased biofilm formation, consistent with the ∆BCAS0292 mutant (Mhatre et al., 2020). Given that the ∆BCAS0292 mutant shares these phenotypes and uniquely expressed RpfR, it is likely that the Bnr1 protein mediates some of its pleiotropic effects via RpfR suppression.

Purified rBnr1 protein forms a dimer that is consistent with many functionally important regulatory proteins, such as transcription factors (Ispolatov et al., 2005; Zhanhua et al., 2005). The unexpected finding that Bnr1 has a highly negatively charged surface indicated that Bnr1 is likely to bind to the nucleic acid‐binding protein(s) and led us to evaluate its impact on DNA topology in the stationary phase. DNA supercoiling is a crude regulator of gene expression in response to environmental signals with the potential to act right across the bacterial genome (Dorman & Corcoran, 2009). The role of DNA supercoiling in global regulation of SPI1 and SPI2 pathogenicity islands genes in *Salmonella enterica* serovar Typhimurium are well described, with negative supercoiling upregulating SPI1 genes and conferring a more invasive phenotype, while SPI2 genes upregulated by DNA relaxation are essential for intramacrophage survival (Croinin et al., 2006; Dorman & Corcoran, 2009). Alterations in *C. jejuni* supercoiling were associated with differences in pathogenesis and an altered secretome (Scanlan et al., 2017). Differences in DNA supercoiling between the ∆BCAS0292 mutant and WT at stationary phase combined with enhanced ciprofloxacin susceptibility reinforced the view that Bnr1 can alter DNA topology, suggesting this is the mechanism by which it wields its regulatory effect.

DNA mimic proteins generally have a molecular weight <25 kDa, pI values <5.0 and are characterized by a negative surface charge distribution, allowing them to mimic the phosphate backbone of DNA (Wang et al., 2019), and Bnr1 matches each of these criteria. Very few (∼20) have been discovered to date because consistent with Bnr1, they have unique sequences and structures and are hard to predict using bioinformatic tools. Half of those identified are bacterial and are involved in stress responses (Wang et al., 2014). Two were identified in *Neisseria* spp., namely DMP12 and DMP19 (Wang et al., 2012, 2013). Like Bnr1, DMP19 forms a homodimer, has a variety of potential binding partners, including a nitrogen response transcription factor (Huang et al., 2017; Wang et al., 2012). DMP12 maintains the stability of unbound HU‐binding regions (Wang et al., 2014, 2013). A DNA mimic in *Mycobacterium tuberculosis*, MfpA, protects the DNA binding site of gyrase from fluoroquinolones (Hegde et al., 2005). Pull‐down analyses to identify possible binding partners of Bnr1 have identified HctB a histone H1‐like protein and a putative histone‐like protein, BCAM1012. A Blast search found no significant similarity between these two proteins (Altschul et al., 1997). HctB is particularly interesting as the *hctB* gene‐encoded Hc2 protein is associated with the transformation of *Chlamydia trachomatis* into the metabolically inactive elementary body in the late stage of its developmental cycle (Barry et al., 1992; Hackstadt et al., 1991). Histone H1‐like proteins, Hc1 and Hc2 are also considered to act as global transcriptional regulators in the transformation of vegetative reticulate bodies into infectious elementary bodies of *Chlamydophila pneumoniae* (Murata et al., 2007). The chlamydial hctB gene caused chromatin condensation and downregulation of transcription when expressed in *E. coli* (Brickman et al., 1993). A histone H1‐like protein with the same H5 signature has also been associated with virulence in *Leishmania* (Papageorgiou & Soteriadou, 2002). Further, the outer membrane localization of HctB may explain, at least in part, the membrane‐associated effects of Bnr1, including polymyxin susceptibility and membrane permeability, Together with the identification of SecA as another binding partner, this supports our initial identification of Bnr1 as an immunogenic protein in people with CF (Shinoy et al., 2013). Future work will involve cloning these proteins to confirm their direct interaction and to elucidate this intriguing mechanism of regulation in more detail.

Overall, we propose that Bnr1, which is dramatically upregulated in response to hypoxic stress interacts with DNA‐binding protein, HctB, altering DNA topology and influencing the expression of genes involved in virulence, host cell attachment, antibiotic susceptibility, and biofilm formation (Figure 7). Thirteen of the *lxa*‐encoded proteins with reduced abundance in the ∆BCAS0292 mutant were also identified in chronic infection isolates suggesting a common adaptive stress response in the hypoxic CF lung (Table S5 at https://doi.org/10.5281/zenodo.5837496). Moreover, 52% of proteins with >1.5 fold‐increased abundance during chronic infection (Cullen et al., 2018) showed >1.5‐reduced abundance in the mutant (Table S6 at https://doi.org/10.5281/zenodo.5837496). Fisher exact test analysis confirmed that these were dependent variables, (Fisher exact test statistic value <0.00001; *p* < 0.05), strengthening the proposed role for Bnr1 in adaptation to chronic infection. Taken together these data suggest that Bnr1 contributes to the adaptation and survival of *B. cenocepacia* in the hypoxic environment of the CF lung.

We propose a mechanism whereby Bnr1 regulates the expression of multiple groups of genes which facilitate its adaptation to stress (Figure 7c). This would allow an environmental pathogen such as *Burkholderia* which is exquisitely successful at thriving in a wide variety of niches from the rhizosphere to the CF lung and disinfectants. The finding that over half of the proteins previously shown to be upregulated in chronic infection isolates (Cullen et al., 2018) are among those of reduced abundance in the mutant suggests that Bnr1 may also play a role in the adaptation to the hypoxic CF lung. Future work will focus on elucidating the mechanism by which Bnr1 and its binding partners mediate this effect.

## CONFLICT OF INTERESTS

None declared.

## ETHICS STATEMENT

None required.

## AUTHOR CONTRIBUTIONS

**Ruth Dennehy**: Conceptualization (equal); investigation (lead); methodology (lead); writing–original draft (equal); writing–review and editing (equal). **Niamh Duggan**: Formal analysis (equal); investigation (supporting); methodology (supporting); writing–review and editing (supporting). **Simon Dignam**: Formal analysis (supporting); investigation (equal); methodology (equal); writing–review and editing (supporting). **Sarah McCormack**: Formal analysis (supporting); investigation (supporting); methodology (supporting). **Eugene Dillon**: Data curation (equal); formal analysis (equal); validation (equal); writing–review and editing (supporting). **Jessica Molony**: Formal analysis (equal); investigation (equal); methodology (supporting). **Maria Romano**: Formal analysis (equal); investigation (equal); methodology (equal); writing–review and editing (supporting). **Yueran Hou**: Formal analysis (equal); investigation (supporting); methodology (supporting); writing–review and editing (supporting). **Laura Ardill**: Methodology (supporting). **Matthew V. X. Whelan**: Methodology (supporting). **Zuzanna Drulis‐Kawa**: Validation (equal); writing–review and editing (supporting). **Tadhg Ó'Cróinín**: Supervision (supporting); validation (equal); writing–review and editing (supporting). **Miguel A. Valvano**: Conceptualization (supporting); methodology (equal); writing–review and editing (supporting). **Rita Berisio**: Conceptualization (supporting); investigation (supporting); software (lead); visualization (supporting); writing–review and editing (supporting). **Siobhán McClean**: Conceptualization (lead); data curation (lead); formal analysis (lead); funding acquisition (lead); investigation (equal); project administration (lead); resources (lead); supervision (lead); visualization (lead); writing–original draft (lead); writing–review and editing (lead).

## Data Availability

The mass spectrometry proteomics data have been deposited to the ProteomeXchange Consortium via the PRIDE partner repository (Perez‐Riverol et al., 2019) with the data set identifiers PXD030042: https://doi.org/10.6019/PXD030042 (whole proteome analysis) and PXD030041: https://doi.org/10.6019/PXD030041 (immunoprecipitation analysis). Data for the Tables S4–S7 are available in the Zenodo repository at https://doi.org/10.5281/zenodo.5837496

## APPENDIX A

See Tables A1, A2 and A3.

**Table A1:** Strains and plasmids used in this study

Strain	Characteristics	Origin
*Burkholderia cenocepacia*		
K56‐2 (LMG18863)	ET12 clone	CF clinical isolate, Canada
Δ*BCAS0292*	Deletion of BCAS0292 in K56‐2	This study
Δ*BCAS0292_0292*	BCAS0292 complement strain; Δ*BCAS0292* chromosomally integrated at the *BCAL1674‐BCAL1675* locus	This study
*Escherichia coli* strains		
NCIB9485		Laboratory strain
GT115	F^−^ *mcrA* Δ(*mrr‐hsd*RMS‐*mcrBC*) Φ80Δ*lac*ZΔM15 Δ*lac*X74 *recA1 rpsL* (StrA) *endA1*Δ*dcm uidA* (ΔMIuI*)::pir‐116* Δ*sbcC‐sbcD*	Invitrogen
SY327	*araD Δ(lac‐pro) argE(Am) recA56 nalA λ pir; Rif* ^*r*^	Miller and Mekalanos (1988)
DH5α	*F* ^−^ *ϕ80dlacZΔM15 Δ(lacZYA‐argF)U169 endA1 recA1 hsdR17(rK* ^−^ *mK* ^+^ *) supE44 thi‐1 ΔgyrA96 relA1*	Invitrogen
Top10	*F* ^−^ *mcrA Δ(mrr‐hsdRMS‐mcrBC) Φ80lacZΔM15 ΔlacΧ74 recA1 araD139 Δ(ara‐leu)7697 galU galK rpsL (Str* ^*R*^ *) endA1 nupG*	Invitrogen
BL21 Star™ (DE3)	F^−^ *ompT hsdS*_B_ (r_B_^−^m_B_^−^) *gal dcm rne131* (DE3)	Invitrogen
Plasmids		
pGPIScel‐2	*ori*_R6K_, ΩTp^r^, mob^+^, containing the ISce‐I restriction site	Flannagan et al. (2008)
pRK2013	*ori*_COIEI_, RK2 derivative, Kan^r^, mob^+^, tra^+^	Figurski and Helinski (1979)
pDAI‐SceI	Encodes the ISce‐I homing endonuclease, Tet^r^	Flannagan et al. (2008)
pMH447	pGPI‐SceI derivative used for chromosomal complementation	Hamad et al. (2012)
pET100/d‐Topo	Peptide fusion at N‐terminal, 6xHis, Amp^r^	Invitrogen
pDA‐12	Reporter plasmid for DNA supercoiling	Aubert et al. (2008)

Abbreviation: CF, cystic fibrosis.

**Table A2:** Primers used in this study

Region	Primer sequence (5′–3′)	Product size (bp)	Restriction enzymes
BCAS0292 gene			
US	actgacgaattccttcgaaaaccaggtggtgtt agtcagatcgatagaccatcgcaacgaacacca	392	*EcoRI ClaI*
DS	gacactatcgatcgatgaaagaccatttctggc cagagtgctagcacacgatcttcagcgtgaact	544	*ClaI NheI*
Internal	cgccagaaatggtctttcat ggtgaccgtgctgttcatc	150	N/A N/A
Sequencing	gttccagatcattgatcgcaaatcaatgcgctgatcgcgaac	534	N/A N/A
Cloning	caccatgtctggtgttcgttgc atccggctacggttcgacgcc	553	N/A N/A
Complementation	ttttcatatgtctggtgttcgttgcgat gttttctagactacggttcgacgccggg	560	*NdeI XbaI*
Plasmids			
pGPI‐Scel‐2	gagtgacacaggaacact gctcaatcaatcaccggatccc	347	N/A N/A
pMH447	ttgatggcgagcgattcttc ccagttcttcagcgtgacga	347	N/A N/A

**Table A3:** Proteins which were unique to either wild‐type k562 or BCAS0292 mutant strain, grouped by function

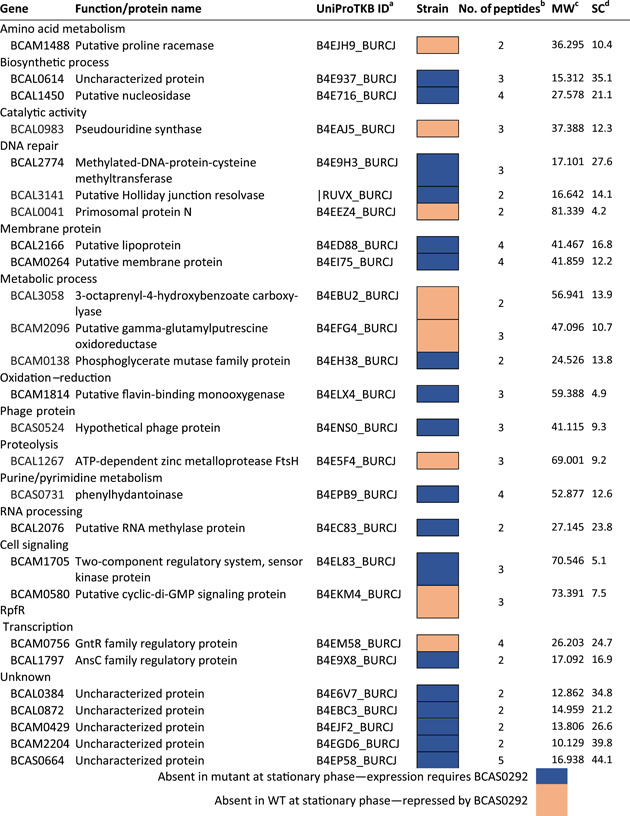

^a^UniProtKB ID Uniprot Knowledge base identifier code.

^b^No. of peptides: Unique peptides: The number of peptide sequences unique to a protein.

^c^Molecular weight as determined by Q‐Exactive LC‐MS using *Burkholderia cenocepacia* J2315 database.

^d^SC: % sequence covered by matching peptides for the identified protein in the database.
